# Farnesol Improves Endoplasmic Reticulum Stress and Hepatic Metabolic Dysfunction Induced by Tunicamycin in Mice

**DOI:** 10.3390/biology14020213

**Published:** 2025-02-18

**Authors:** Naqash Goswami, Lionel Kinkpe, Lun Hua, Yong Zhuo, Zhengfeng Fang, Lianqiang Che, Yan Lin, Shengyu Xu, Xuemei Jiang, Bin Feng, De Wu

**Affiliations:** 1 Animal Nutrition Institute, Sichuan Agricultural University, Chengdu 611130, China; goswaminaqash@gmail.com (N.G.); hualun@sicau.edu.cn (L.H.); zhuoyong@sicau.edu.cn (Y.Z.); zfang@sicau.edu.cn (Z.F.); chelianqiang@sicau.edu.cn (L.C.); linyan@sicau.edu.cn (Y.L.); shengyux@sicau.edu.cn (S.X.); 71310@sicau.edu.cn (X.J.); 2 College of Animal Science and Technology, Northwest Agriculture and Forestry University, Yangling 712100, China; klionel@nwsuaf.edu.cn

**Keywords:** endoplasmic reticulum stress, hepatic energy metabolism, oxidative stress

## Abstract

Endoplasmic reticulum (ER) stress significantly affects liver metabolism, often leading to disorders such as hepatic steatosis. Tunicamycin (TM), a known ER stress inducer, is frequently used to model metabolic stress, but its specific effects on liver energy homeostasis remain unclear. This study investigates how farnesol (FOH), a natural compound with antioxidant and anti-inflammatory properties, counteracts TM-induced ER stress and its associated metabolic disruptions in the liver. Using both primary hepatocytes and a mouse model, this study demonstrates that TM treatment caused upregulation of ER stress markers, including ATF4, and disrupted genes related to lipid metabolism and gluconeogenesis. Co-treatment with FOH reduced these stress markers and restored the expression of metabolic genes. In vivo, FOH treatment alleviated oxidative stress, reduced lipid accumulation, and restored normal glycogen and lipid metabolism. Histological analysis further confirmed that FOH preserved liver architecture and minimized cellular damage. FOH also stabilized serum lipid profiles and modulated key metabolic biomarkers, suggesting its protective role against TM-induced liver injury. These findings suggest that FOH has therapeutic potential in mitigating ER stress-related metabolic dysfunctions, offering promising insights for the treatment of liver diseases linked to metabolic stress.

## Simple Summary

This study investigated the potential of farnesol (FOH), a natural compound found in essential oils, to protect the liver from stress induced by tunicamycin (TM), a substance known to disrupt cellular processes and contribute to liver diseases. TM causes oxidative stress, lipid accumulation, and alters liver function, leading to metabolic disorders such as fatty liver disease. In this study, both liver cells and animal models were treated with TM to simulate these conditions. The findings show that FOH could reduce the harmful effects of TM, restoring normal liver function, reducing fat accumulation, and preserving healthy liver structure. This study highlights FOH’s potential as a natural therapy for liver diseases linked to metabolic stress.

## 1. Introduction

The liver, a central organ in metabolism, plays a vital role in maintaining systemic energy homeostasis, including glucose regulation, lipid metabolism, and glycogen synthesis [1]. It manages key metabolic processes such as gluconeogenesis, lipogenesis, and fatty acid oxidation, all of which are regulated by various enzymes and influenced by insulin signaling and endoplasmic reticulum (ER) stress [2,3,4]. ER stress occurs when the folding capacity of the ER is overwhelmed, leading to the accumulation of misfolded proteins and triggering the unfolded protein response (UPR) [5]. In fact, the ER is a critical organelle involved in protein synthesis, post-translational modification, and trafficking, and is highly sensitive to stressors that disrupt cellular homeostasis. These stressors, which include disruptions in energy levels, redox state, and calcium ion (Ca^2+^) concentrations, can impair the ER’s ability to correctly fold and process proteins. As a result, misfolded or unfolded proteins accumulate within the ER lumen, triggering a protective cellular mechanism known as the UPR [6].

The primary goal of the UPR is to restore ER function and cellular homeostasis by enhancing protein folding capacity, halting protein synthesis, and facilitating the degradation of misfolded proteins. However, if ER stress persists, it can lead to the chronic activation of the UPR [7,8]. The UPR is initiated through the activation of three key signaling pathways: IRE1 (Inositol-requiring enzyme 1), PERK (protein kinase RNA-like ER kinase), and ATF6 (activating transcription factor 6). Each of these pathways plays a distinct role in alleviating ER stress [9,10]. Collectively, PERK, ATF6, and IRE1α initiate separate branches of the UPR [11]. Therefore, the ATF4 is central to all branches of the integrated stress response, but it is only involved in 1 of the 3 branches of the UPR. Unlike other branches of the UPR, which primarily focus on protein folding and quality control, ATF4 plays a unique role in modulating the cellular response to oxidative damage by activating genes that counteract the harmful effects of reactive oxygen species (ROS).

Consequently, understanding the dual nature of the UPR, particularly through the actions of ATF4, is crucial for identifying potential therapeutic targets in stress-related diseases, including metabolic diseases and liver-related pathologies like non-alcoholic fatty liver disease (NAFLD), alcoholic liver disease (ALD) [12], neurodegenerative diseases [10], and cardiovascular diseases [13]. While the understanding of cellular stress responses sheds light on the pathogenesis of various ailments, recent advancements have also emphasized the potential of natural compounds in mitigating stress-related health concerns.

Farnesol (FOH), a sesquiterpene alcohol found in essential oils, has shown considerable promise due to its anti-inflammatory, antioxidant, and anti-cancer properties [14,15]. Previous studies pointed out FOH’s potential to combat oxidative stress [16,17], inflammation [17], and metabolic disorders [18,19]. Given its broad pharmacological effects and low cytotoxicity [20], FOH is emerging as a therapeutic candidate for conditions associated with ER stress.

One of the well-known inducers of ER stress is tunicamycin (TM), which disrupts N-linked glycosylation in proteins, leading to the accumulation of misfolded proteins in the ER and the activation of UPR [21]. TM can also stimulate tumor cell apoptosis [22], and it has been used to develop an anti-cancer drug [23,24]. Furthermore, due to its capacity to induce ER stress, TM has been shown to disrupt metabolic pathways, such as inhibiting the phosphorylation of protein kinase B (Akt) in hepatocytes, which is crucial for insulin sensitivity and the regulation of glucose and triglyceride metabolism [25]. However, short-term exposure to TM has been shown to suppress hepatic gluconeogenesis [26] and promote lipid accumulation [27], suggesting its significant impact on liver metabolism and fat storage.

Additionally, research has demonstrated that TM induces triglyceride accumulation in cultured HepG2 hepatic cells, suggesting its role in disrupting lipid regulation [28]. TM has been shown to induce hepatic ER stress, increasing triglyceride accumulation and decreasing glycogen content, thereby disrupting liver homeostasis. It also alters serum lipid profiles by lowering triglycerides, LDL, HDL, and apolipoprotein B levels, while suppressing apolipoprotein B100 expression and protein kinase B signaling in the liver, demonstrating its complex impact on both hepatic and systemic lipid metabolism [29].

Despite the growing body of research on the effects of tunicamycin (TM) on liver function, the protective role of farnesol (FOH) in mitigating these effects has not been well explored. This study aims to fill this gap by investigating the potential of FOH in alleviating TM-induced ER stress, oxidative stress, and hepatic metabolic disruptions, using primary hepatocytes and animal models to evaluate its efficacy in restoring normal hepatic function under stress.

Results indicated that as compared with the control group, farnesol treatment under TM-induced ER stress significantly reduced ER stress markers such as ATF4, restored metabolic gene expression (SREBP1c, CPT1A, PEPCK), improved antioxidant indices (SOD, GSH), mitigated lipid droplet accumulation, and stabilized serum lipid profiles, suggesting its potential as a therapeutic agent for metabolic disorders linked to ER stress.

## 2. Materials and Methods

### 2.1. Animal Experiments

The animal study protocol was reviewed and approved by the Animal Care and Use Committee of Sichuan Agricultural University. All animal procedures were performed according to the National Institutes of Health guide for the care and use of laboratory animals. Healthy C57BL/6 male mice (2 months old, 20–23 g) were obtained from Dashuo Biotechnology Co., Ltd. (Chengdu, China) and maintained under specific pathogen-free conditions at 22 ± 1 °C on a 12:12 h light/dark cycle with free access to food and water. The mice were allowed one week of acclimation in a pathogen-free room at humidity of 60%. Subsequently, they were randomly assigned to three groups (*n* = 8 per group): control (vehicle 0.5 mg/kg body weight, BW), tunicamycin-treated (TM 0.5 mg/kg BW), and co-treatment with farnesol and tunicamycin (FOH+TM 20 mg/kg BW). All treatments were administered via intraperitoneal injection, and after 6 h, mice were euthanized by CO_2_ asphyxiation. Blood was collected via cardiac puncture, and tissues, including liver and epididymal fat, were excised, weighed, and either fixed in 10% formalin for histological analysis or frozen in liquid nitrogen for further biochemical studies. Serum was isolated from blood after centrifugation at 4 °C, 3000 rpm for 15 min and stored at −80 °C.

### 2.2. Cell Model and Reagents

The primary hepatocytes used in this study were isolated from male mice (8–10 weeks old, weighing 200–250 g) using a well-established collagenase-based perfusion technique, as described by [26]. Briefly, the liver was perfused with a collagenase solution (Sigma-Aldrich, Darmstadt, Germany), and after enzymatic dissociation, the liver was mechanically disrupted to release hepatocytes. These cells were then collected, washed, and resuspended in complete culture medium. The hepatocytes were cultured in Dulbecco’s Modified Eagle Medium (DMEM) containing 10% fetal bovine serum (FBS), 100 U/mL penicillin, and 100 μg/mL streptomycin under standard conditions at 37 °C with 5% CO_2_.

To assess the purity of the isolated hepatocyte fraction, we employed a combination of morphological analysis and flow cytometry. Morphologically, hepatocytes were examined for characteristic polygonal shapes, indicative of mature hepatocytes, and flow cytometry was performed using specific markers such as cytochrome P450 and albumin to confirm hepatocyte identity, with CD45 serving as a marker for non-parenchymal cells. The purity of the isolated hepatocyte fraction was consistently over 90%, with minimal contamination from non-parenchymal cells. This high level of purity ensures that the observed effects in our experiments are primarily due to hepatocytes and not influenced by non-parenchymal cells, thereby ensuring the reliability of our findings. Cells were maintained at 90% confluence, detached using 0.25% trypsin for 20–30 s at room temperature, and replated into 12-well plates at 60% confluence. Following that, cells were incubated overnight in serum-free DMEM (SF-DMEM) and coated with type-I collagen. All reagents, including farnesol and tunicamycin, were sourced from Sigma-Aldrich (Darmstadt, Germany) or Fisher Scientific (Thermo Fisher Scientific, Waltham, MA, USA) unless otherwise stated (Supplementation Materials Tables S1 and S2).

### 2.3. Cell Culture and Treatment

In this study, hepatocytes were treated with various concentrations of farnesol (FOH) (10, 25, 30, 50, and 100 µM) for 6, 12, and 24 h to determine the optimal dose and treatment duration for evaluating the protective effects against tunicamycin (TM)-induced endoplasmic reticulum (ER) stress. After overnight incubation in serum-free DMEM, cells were exposed to TM (1 µg/mL) for 6 h to induce ER stress, and the effects of FOH on oxidative stress and metabolic disturbances were assessed. Our preliminary experiments showed that FOH at 25 µM for 6 h significantly mitigated TM-induced ER stress without cytotoxicity, as confirmed by cell viability assays. Cell viability was assessed using the trypan blue dye exclusion method. Cells were mixed with trypan blue dye and placed onto a hemocytometer (a slide divided into four large sections). The hemocytometer’s grid enabled precise counting of cells, where live cells, which have intact membranes, excluded the dye, and dead cells took up the dye due to compromised membranes. The slide was then examined under an electron microscope, and the ratio of live to dead cells was determined by counting the number of viable cells (those that did not uptake the dye) versus those that did. This early protective effect was evident at the 6-h mark, suggesting that this time point was sufficient to capture the acute therapeutic benefits of FOH. Based on these results, 25 µM FOH for 6 h was selected as the optimal treatment for further experiments to assess the early-phase protective effects of FOH. The 6-h endpoint was chosen because significant cellular responses, including oxidative stress and metabolic disturbances, were detectable within this time frame, consistent with the activation of stress response pathways such as the unfolded protein response (UPR), which is central to TM-induced toxicity. While TM-induced damage continues over 24 to 48 h, our primary aim was to investigate the immediate cellular response to TM exposure, and the 6-h period allowed us to evaluate the potential of FOH as an early intervention to restore cellular homeostasis during the initial phase of ER stress. After the treatment period, the medium was discarded, and cells were washed twice with phosphate-buffered saline (PBS) before harvesting for RNA extraction.

### 2.4. RNA Isolation, cDNA Synthesis, and qPCR

Total RNA was isolated from hepatocytes and liver tissue using TRIzol reagent (Thermo Fisher Scientific, Waltham, MA, USA) and purified with the RNase Mini Kit (Takara, San Jose, CA, USA), following the manufacturer’s instructions. After treating the cells with 25 µM farnesol (FOH) and 0.5 µg/mL tunicamycin (TM) for 6 h, the cells were washed twice with PBS, and RNA was extracted. One microgram of RNA was reverse-transcribed into complementary DNA (cDNA) using a reverse transcription PCR kit. Quantitative PCR (qPCR) was then performed using SYBR Green reagents on a qPCR system (Thermo Fisher Scientific, Waltham, MA, USA). RNA concentration was determined using a Nanodrop spectrophotometer (Thermo Fisher Scientific, Waltham, MA, USA). Gene expression was quantified using the 2^−ΔΔCt^ method. The amounts of reagents used for each reaction are summarized in Supplementary Tables S3 and S4, and the primer sequences used in the study are detailed in Supplementary Table S5.

### 2.5. Assessment of Blood Chemistry

Healthy C57BL/6 male mice were euthanized via abdominal incision, and blood samples were collected. Serum was isolated by centrifugation at 4 °C and 3000 rpm for 15 min. Serum biochemical markers were analyzed, including aspartate transaminase (AST), alanine transaminase (ALT), free fatty acids (NEFA), total cholesterol (TC), triglycerides (TAG), glycogen, high-density lipoprotein cholesterol (HDL-C), and low-density lipoprotein cholesterol (LDL-C), using assay kits from Makerbio Co. (Chengdu, China) according to the manufacturer’s instructions. The measurements were performed with a HITACHI biochemical analyzer (Model 7020, Tokyo, Japan).

### 2.6. Assessment of Liver Glycogen and Triglyceride Content

Liver glycogen and triglyceride contents were quantified using the Nanjing Completion Kit. For glycogen analysis, 100 mg of liver tissue was homogenized with alkaline solution (3 times the tissue weight), boiled for 20 min, diluted, and centrifuged at 12,000 rpm for 5 min. A 200 µL aliquot of the supernatant was mixed with 400 µL of color reagent, incubated at 98 °C for 5 min, and absorbance was measured at 610 nm. Glycogen content was calculated using a standard curve. For triglycerides, 50 mg of liver tissue was homogenized with ethanol (30 times the tissue weight), mixed, and centrifuged at 12,000 rpm for 5 min. The supernatant was transferred, and 5 µL of the sample was added to a 96-well plate with 200 µL of enzyme solution. The samples were incubated at 37 °C for 10 min, and absorbance was measured at 510 nm to determine triglyceride content.

### 2.7. Biochemical Index Determination of MDA, SOD, and GSH in Liver Tissue

To assess oxidative stress in the liver, levels of malondialdehyde (MDA), superoxide dismutase (SOD), and glutathione (GSH) were quantified, given their respective roles in lipid peroxidation, enzymatic antioxidant defense, and redox homeostasis. Liver tissues were rinsed with ice-cold saline, flash-frozen, and stored at −80 °C until analysis. Homogenization was performed at 4 °C using pre-cooled 0.9% saline (1:9, *w*/*v*), followed by centrifugation at 10,000× *g* for 10 min at the same temperature. The supernatant was collected for biochemical analyses, with protein concentrations determined using the BCA Protein Assay Kit (Thermo Fisher Scientific, Cat. No. 23225) as per the manufacturer’s protocol.

MDA levels were measured using the Lipid Peroxidation MDA Assay Kit (Nanjing Jiancheng Bioengineering Institute, Nanjing, China), based on the thiobarbituric acid reactive substances (TBARS) method. Briefly, 0.2 mL of the supernatant was mixed with 1 mL of the MDA reagent, incubated at 95 °C for 60 min, cooled to room temperature, and the absorbance was recorded at 532 nm. The concentration was extrapolated from a standard curve and expressed as nmol/mg protein. GSH quantification was performed using the GSH Assay Kit (Nanjing Jiancheng Bioengineering Institute), where 0.1 mL of the supernatant was incubated with 1 mL of the reaction buffer at 37 °C for 10 min, and absorbance was measured at 405 nm, with results expressed as µmol/mg protein.

SOD activity was assessed using the Superoxide Dismutase Assay Kit (Nanjing Jiancheng Bioengineering Institute), which quantifies the inhibition of nitroblue tetrazolium (NBT) reduction. A 0.2 mL aliquot of the supernatant was reacted with the assay reagents, and absorbance was measured at 550 nm. Activity was calculated based on a standard curve and expressed as U/mg protein.

### 2.8. Histological and Ultrastructure Analysis

Histopathological analysis was performed to assess liver injury induced by tunicamycin and the protective effect of farnesol. Liver samples were fixed in 4% paraformaldehyde for 24 h, followed by routine paraffin embedding. Tissue sections (5 μm thick) were cut and stained with hematoxylin and eosin (H&E) to evaluate hepatic injury. Images were captured using a Nikon Eclipse 50i digital camera (TS100, Tokyo, Japan) for further assessment. A pathologist, blinded to the treatment groups, graded the severity of liver injury. The histological sections were analyzed using a light microscope, and scoring was conducted for a quantifiable evaluation of liver damage.

### 2.9. Statistical Analysis

Data were recorded using Microsoft Excel® and analyzed with R 4.3.2 software (R Core Team, 2023). Analysis of variance (ANOVA) was employed to compare means. Graphs were constructed using GraphPad Prism 6 (GraphPad Software, San Diego, CA, USA). Statistical significance was determined at *p* < 0.05.

## 3. Results

**FOH Ameliorates Tunicamycin-Induced Oxidative Stress and Altered Hepatic Energy Homeostasis**

### 3.1. Farnesol Reduces Tunicamycin-Induced ER Stress by Downregulating Key Stress Markers in Primary Hepatocytes

**Figure 1:**
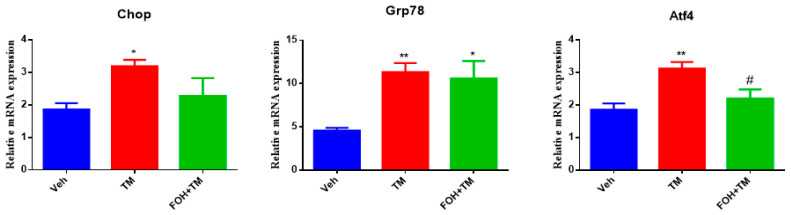
Ameliorative action of farnesol against tunicamycin-induced changes in relative expression of the endoplasmic reticulum stress-related genes Chop, Grp78, and Atf4 in primary hepatocytes. Values represent the mean ± SEM from three independent biological replicates. Statistical significance was determined by one-way ANOVA followed by Tukey’s multiple comparison test. Veh: Control (vehicle); TM: Tunicamycin; FOH+TM: Farnesol plus tunicamycin. * *p* < 0.05, ** *p* < 0.01, vs. Veh, # *p* < 0.05 vs. TM.

To evaluate the effect of farnesol (FOH) on tunicamycin (TM)-induced endoplasmic reticulum stress (ERS) in primary hepatocytes, a preliminary study was conducted using five different concentrations of FOH (10, 25, 30, 50, and 100 µM) to determine the optimal dose for further experiments. At a concentration of 25 µM, FOH exhibited no cytotoxicity toward primary hepatocytes. The expression levels of ER stress markers were measured using qRT-PCR, and it was found that FOH effectively inhibited TM-induced ER stress, which typically leads to the upregulation of genes such as Chop, Grp78, and Atf4 (Figure 1).

The time course of mRNA expression for these ER stress markers in response to TM treatment was examined. After 6 h of TM exposure, a significant increase in the mRNA levels of Grp78, Chop, and Atf4 was observed compared to the control group (treated with DMSO). Treatment with FOH at 25 µM significantly reduced the mRNA expression of Grp78 and Atf4, indicating a mitigation of ER stress in hepatocytes. However, the expression of Chop remained relatively unaffected by FOH treatment.

These findings demonstrate that FOH can inhibit ER stress in primary hepatocytes by reducing the expression of key ER stress markers, although its effect on Chop appears limited. This suggests that FOH has potential as a modulator of ER stress, providing protection against TM-induced cellular stress responses.

### 3.2. Farnesol Neutralized Metabolic Gene Expression and Ameliorates Tunicamycin-Induced Hepatic Energy Disruptions

**Figure 2:**
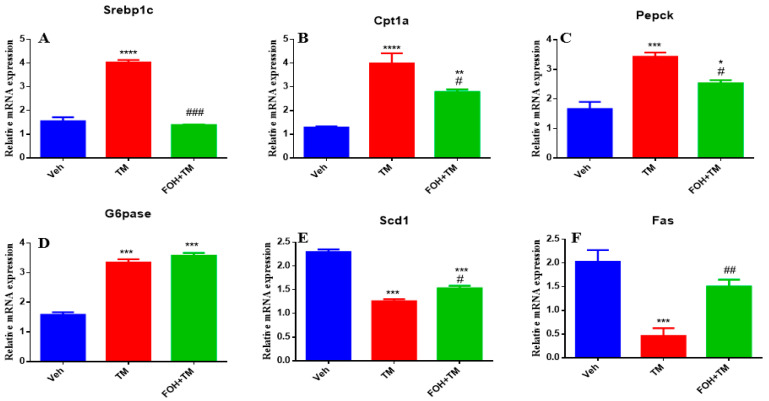
Ameliorative effect of farnesol on tunicamycin-induced changes in the relative expression of hepatic energy homeostasis-related genes. (**A**) Gene expression of Srebp1c; (**B**) Gene expression of Cpt1a; (**C**) Gene expression of Pepck; (**D**) Gene expression of G6pase; (**E**) Gene expression of Scd1; (**F**) Gene expression of Fas in primary hepatocytes. Values represent the mean ± SEM from three independent biological replicates. Veh: control (vehicle); TM: tunicamycin; FOH+TM: farnesol plus tunicamycin. * *p* < 0.05, ** *p* < 0.01, *** *p* < 0.001, **** *p* < 0.0001 vs. Veh; # *p* < 0.05, ## *p* < 0.01, ### *p* < 0.001 vs. TM.

Farnesol (FOH) was not cytotoxic to primary mouse hepatocytes at a concentration of 25 µM. To investigate its effect on tunicamycin (TM)-induced disturbances in hepatic energy homeostasis, primary hepatocytes were incubated with or without FOH for 6 h. The results demonstrated that TM significantly (*p* < 0.05) altered the mRNA expression of key metabolic genes, including Srebp1c, Cpt1a, Pepck, and G6pase. Co-treatment with FOH significantly (*p* < 0.05) neutralized the expression of Srebp1c, Cpt1a, and Pepck, but did not affect G6pase expression (Figure 2).

Furthermore, TM treatment significantly (*p* < 0.05) decreased the mRNA levels of Scd1 and Fas, while co-treatment with FOH effectively reversed this reduction. These findings indicate that FOH ameliorates TM-induced disruptions in hepatic energy metabolism by modulating the expression of key metabolic genes, suggesting its potential protective role against ER stress-induced hepatic dysfunction.

### 3.3. Farnesol Selectively Reduces Atf4 Expression in Tunicamycin-Induced ER Stress

**Figure 3:**
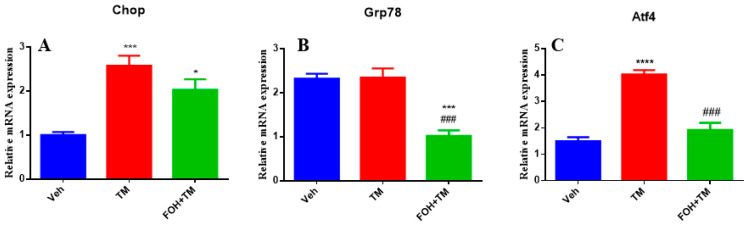
Ameliorative action of farnesol against tunicamycin-induced alterations in the relative expression of endoplasmic reticulum stress (ERS)-related genes. (**A**) Gene expression of Chop; (**B**) Gene expression of Grp78; (**C**) Gene expression of Atf4 in the liver tissue of mice. Values represent the mean ± SEM from eight biological replicates per group. Veh: control (vehicle); TM: tunicamycin; FOH+TM: farnesol plus tunicamycin. *p < 0.05, *** *p* < 0.001 vs. Veh; **** *p* < 0.0001 vs. Veh; ### *p* < 0.001 vs. TM.

To evaluate the effects of FOH on TM-induced hepatic ER stress, RT-qPCR analysis was performed to measure the mRNA expression of the key ER stress markers Chop, Grp78, and Atf4 in liver tissue (Figure 3). TM treatment significantly (*p* < 0.05) upregulated the expression of all three markers, indicating activation of ER stress pathways. Co-treatment with FOH+TM led to a significant (*p* < 0.05) reduction in Atf4 expression, suggesting that FOH alleviates some aspects of ER stress. However, FOH did not significantly alter the mRNA levels of Chop and Grp78 compared to the TM-treated group. These findings indicate that FOH selectively mitigates ER stress by downregulating Atf4, while its impact on Chop and Grp78 remains limited.

### 3.4. Farnesol Restores Hepatic Metabolic Gene Expression in Tunicamycin-Induced ER Stress

**Figure 4:**
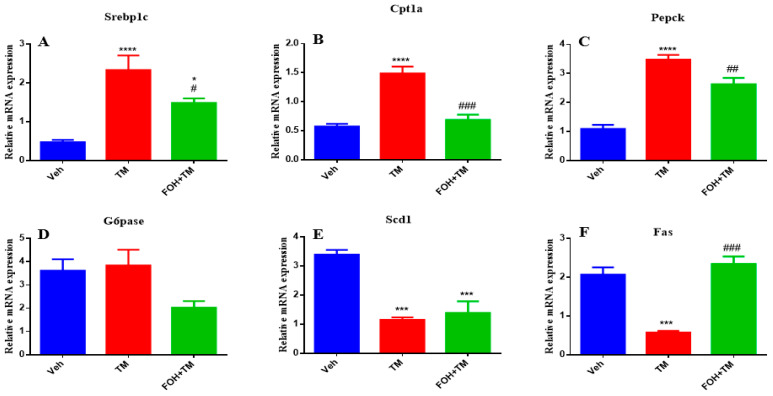
Ameliorative effect of farnesol on tunicamycin-induced alterations in the relative expression of metabolic genes. (**A**) Gene expression of Srebp1c; (**B**) Gene expression of Cpt1a; (**C**) Gene expression of Pepck; (**D**) Gene expression of G6pase; (**E**) Gene expression of Scd1; (**F**) Gene expression of Fas in liver tissue. Values represent the mean ± SEM from eight biological replicates per group. Veh: Control (vehicle); TM: Tunicamycin; FOH+TM: Farnesol plus tunicamycin. * *p* < 0.05, *** *p* < 0.001, **** *p* < 0.0001 vs. Veh; # *p* < 0.05, ## *p* < 0.01, ### *p* < 0.001 vs. TM.

To assess the positive effects of farnesol (FOH) on tunicamycin (TM)-induced alterations in hepatic homeostasis, the mRNA expression of key metabolic genes was measured (Figure 4). TM treatment significantly (*p* < 0.05) increased the expression of Srebp1c, Cpt1a, and Pepck in liver tissue compared to the control group. Co-treatment with FOH+TM significantly (*p* < 0.05) reduced the expression of these genes, restoring them to near control levels. However, the expression of G6pase was not significantly altered by either TM or FOH+TM treatment.

Similarly, TM treatment significantly (*p* < 0.05) downregulated Scd1 and Fas expression, while co-treatment with FOH+TM significantly improved their expression (*p* < 0.05 *), although the increase in Scd1 did not reach statistical significance. These results suggest that FOH has the ability to restore hepatic homeostasis by regulating the expression of key metabolic genes affected by TM-induced ER stress.

### 3.5. Farnesol Alleviates Tunicamycin-Induced Oxidative Stress by Enhancing Antioxidant Defense in the Liver

**Figure 5:**
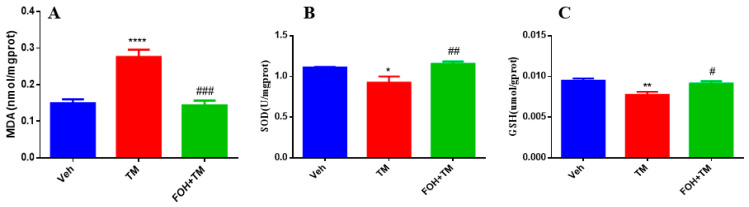
Protective effect of farnesol against tunicamycin-induced oxidative stress in the liver. (**A**) MDA levels; (**B**) SOD activity; (**C**) GSH levels. Values represent the mean ± SEM from eight biological replicates per group. Statistical significance was determined using one-way ANOVA followed by Tukey’s multiple comparison test. Veh: Control (vehicle); TM: Tunicamycin; FOH+TM: Farnesol plus tunicamycin. * *p* < 0.05, ** *p* < 0.01, **** *p* < 0.0001 vs. Veh; # *p* < 0.05, ## *p* < 0.01, ### *p* < 0.001 vs. TM.

To evaluate the protective effects of FOH against TM-induced oxidative stress in the liver, antioxidant enzyme levels of MDA, SOD, and GSH were measured (Figure 5). TM treatment significantly (*p* < 0.05) elevated hepatic MDA levels, indicating increased lipid peroxidation and oxidative stress. In contrast, co-treatment with FOH+TM restored MDA levels, mitigating the oxidative damage induced by TM.

Additionally, the levels of SOD and GSH, key antioxidant defenses, were significantly reduced in the TM-treated group. However, co-treatment with FOH+TM significantly improved both SOD and GSH levels, restoring them to values comparable to the control group. These findings suggest that FOH effectively reduces TM-induced oxidative stress in the liver by enhancing antioxidant enzyme activity and reducing lipid peroxidation.

Following the above results, this in vivo session aims to focus on how FOH affects physical, histological, and biochemical changes in the liver in response to tunicamycin-induced damage.

### 3.6. Farnesol Protects Against Tunicamycin-Induced Hepatic Injury and Lipid Accumulation

**Figure 6:**
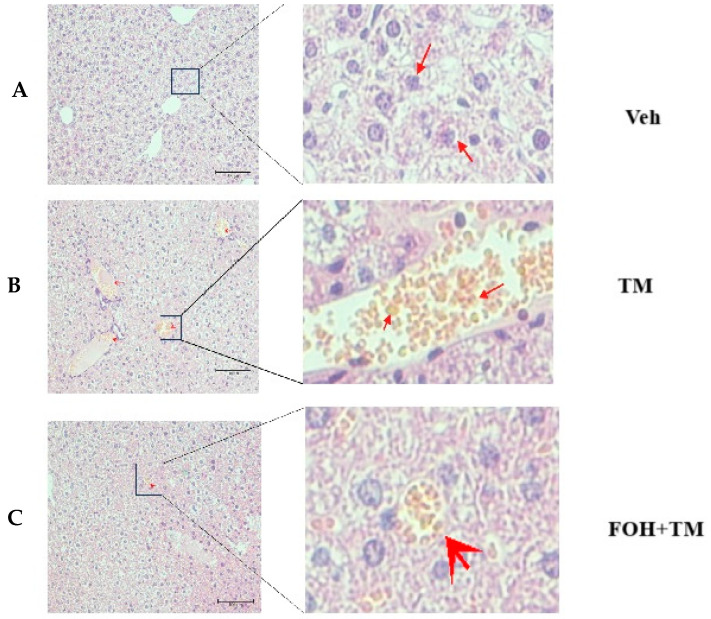
Histopathological and ultrastructural changes demonstrated by liver tissue of mice in hematoxylin and eosin staining after exposure to tunicamycin. (**A**) Control (Veh) group histological examination of H&E-stained. (**B**) Tunicamycin (TM) group histological examination of H&E-stained. (**C**) Farnesol plus tunicamycin (FOH+TM) group histological examination of H&E-stained. Note: The farnesol-treated group significantly improves the health of liver tissue of mice when compared with the tunicamycin-treated and control groups of experimental mice.

To evaluate the protective effect of farnesol (FOH) on tunicamycin (TM)-induced hepatic injury, liver histological and ultrastructural analyses were performed (Figure 6). In the TM-treated group, histological assessment revealed a significant accumulation of enlarged hepatic lipid droplets compared to the control (Veh) and FOH+TM co-treated groups. Treatment with FOH+TM significantly (*p* < 0.05) reduced lipid droplet accumulation, with smaller and fewer droplets observed compared to the TM group.

Ultrastructural analysis further supported these findings, showing irregular, fragmented, and condensed nuclei as well as swollen hepatocytes in the TM-treated group. These abnormalities were absent in the control group, which displayed normal liver architecture. Additionally, vacuoles associated with hepatocyte inflammation were observed in some areas of the TM-treated liver under electron microscopy. In contrast, FOH+TM co-treatment improved the overall liver structure, suggesting that FOH mitigates TM-induced hepatic lipid accumulation and cellular damage.

### 3.7. Farnesol Restores Glycogen and Lipid Metabolism in Tunicamycin-Induced Hepatic Dysfunction

**Figure 7:**
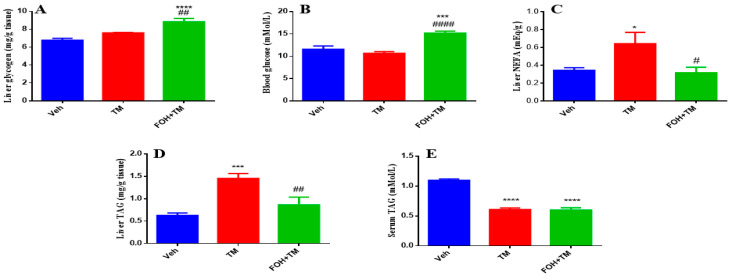
The ameliorative action of farnesol against tunicamycin-induced alterations. (**A**) Liver glycogen levels; (**B**) Blood glucose levels; (**C**) Non-esterified fatty acid levels; (**D**) Liver TAG levels; (**E**) Serum TAG levels. Values represent the mean ± SEM from eight independent biological replicates. Control (Veh), tunicamycin (TM), and farnesol plus tunicamycin (FOH+TM) treatment groups. * *p* < 0.05, *** *p* < 0.001, **** *p* < 0.0001 vs. Veh; # *p* < 0.05, ## *p* < 0.01, #### *p* < 0.0001 vs. TM.

To evaluate the effects of FOH on TM-induced alterations in hepatic glycogen and triglyceride content, both serum and liver tissue samples were analyzed (Figure 7). Liver glycogen levels show no significant change following TM treatment, but FOH co-treatment restores glycogen content to baseline levels, indicating that FOH helps maintain glycogen storage in the face of potential metabolic disruptions. Similarly, blood glucose levels are not significantly altered by TM, though FOH co-treatment significantly reduces blood glucose, suggesting that FOH may have a regulating effect on glucose levels. Liver non-esterified fatty acids (NEFA) significantly increase in the TM-treated group, and FOH co-treatment significantly reduces these levels, indicating protection against lipid accumulation. Liver triglyceride (TAG) content increases significantly after TM treatment, and FOH co-treatment reduces TAG levels, indicating that FOH alleviates hepatic steatosis. Lastly, serum TAG levels are significantly elevated in the TM group and reduced by FOH, supporting the idea that FOH helps restore normal lipid metabolism. Overall, FOH effectively mitigates TM-induced metabolic disturbances by reducing lipid accumulation in the liver and serum, without significant effects on glycogen and blood glucose.

### 3.8. Farnesol Modulates Serum Biomarkers in Tunicamycin-Induced Hepatic Stress

**Figure 8:**
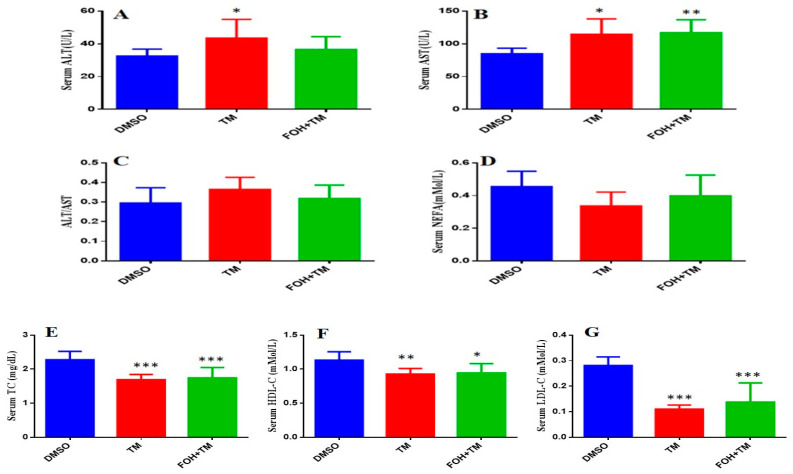
Ameliorative action of farnesol against tunicamycin-induced alterations in blood serum markers. (**A**) Serum ALT levels; (**B**) Serum AST levels; (**C**) ALT/AST ratio; (**D**) NEFA levels; (**E**) Serum TC levels; (**F**) Serum HDL-C levels; (**G**) Serum LDL-C levels. Values represent the mean ± SEM from eight independent biological replicates. Control (Veh), tunicamycin (TM), and farnesol plus tunicamycin (FOH+TM) treatment groups. * *p* < 0.05, ** *p* < 0.01, *** *p* < 0.001 vs. Veh.

The serum biomarkers of experimental mice were measured using an automatic biochemical analyzer (7020, HITACHI, Tokyo, Japan) with respective analysis kits (Figure 8). TM treatment significantly (*p* < 0.05) elevated aspartate transaminase (AST) and alanine transaminase (ALT) levels, indicating liver damage, while the AST/ALT ratio remained unchanged. Co-treatment with FOH+TM did not significantly alter AST or ALT levels compared to the TM group.

In contrast, TM-treated mice showed significant (*p* < 0.05) reductions in total cholesterol (TC), high-density lipoprotein cholesterol (HDL-C), and low-density lipoprotein cholesterol (LDL-C) levels compared to controls. Although the FOH+TM group showed improvement in these markers, the changes did not reach statistical significance. The outcome of this may suggest that while FOH may influence lipid metabolism, its effects on serum biomarkers in TM-induced hepatic stress require further investigation.

### 3.9. Farnesol Mitigates Tunicamycin-Induced Changes in Liver and Fat Accumulation Without Affecting Body Weight

**Figure 9:**
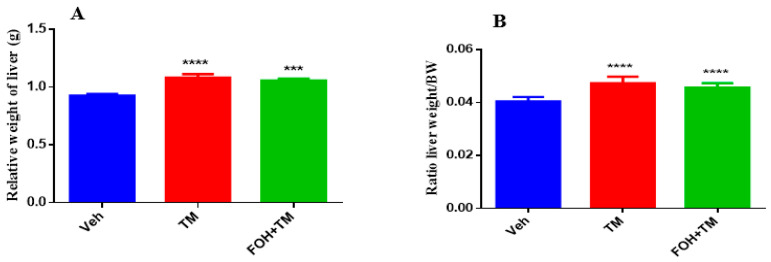
Ameliorative action of farnesol against tunicamycin-induced alterations in liver weight parameters. (**A**) Relative weight of liver; (**B**) Liver weight/BW ratio in experimental mice. Values represent the mean ± SEM from eight independent biological replicates. Control (Veh), tunicamycin (TM), and farnesol plus tunicamycin (FOH+TM) treatment groups. *** *p* < 0.001, **** *p* < 0.0001 vs. Veh.

The effects of farnesol on body weight, epididymal fat weight, and liver index measurements are presented in Figure 9. TM treatment significantly increased body and epididymal fat weight compared to controls, with no significant differences observed between the TM and FOH+TM groups. Liver weight and the liver-to-body weight ratio also significantly changed in the TM group, with no morphological abnormalities in the FOH+TM group compared to the yellowish liver appearance in the TM group.

## 4. Discussion

This study explores the potential of farnesol (FOH) in alleviating oxidative stress and restoring metabolic homeostasis in liver cells subjected to tunicamycin (TM)-induced endoplasmic reticulum (ER) stress. The growing interest in plant-based compounds, particularly FOH, stems from its reported protective effects against oxidative stress and inflammation, which are commonly implicated in metabolic disorders [30]. Previous research has highlighted the potential of farnesol (FOH) in addressing various health concerns, including its protective effects against oxidative stress, inflammation, and metabolic disorders [19], yet its specific effects on TM-induced ER stress in hepatocytes have remained underexplored. Our findings fill this gap by demonstrating that FOH significantly mitigates TM-induced ER stress and restores hepatic metabolic function.

FOH achieves this through several mechanisms. First, it downregulates key ER stress markers, particularly ATF4, which plays a central role in the UPR. In fact, ATF4 is crucial for cellular adaptation under stress by regulating genes involved in amino acid metabolism, redox balance, and cell survival [31,32]. Dysregulation of ATF4 often leads to apoptosis, underscoring its dual role in determining cell fate under stress [33]. Our study confirms that FOH reduces ATF4 expression, enhancing cellular resilience against stress and protecting against liver injury. By reducing ATF4 expression, FOH may enhance cellular resilience against TM-induced stress, supporting its potential as a protective agent in hepatic injuries [34]. This observation aligns with earlier studies [35,36,37], which suggest that modulating ATF4 could be a promising therapeutic strategy in liver diseases associated with ER stress.

In addition to its effects on ER stress, FOH restores metabolic gene expression, including SREBP1c, CPT1A, and PEPCK, which are essential for lipid synthesis, fatty acid oxidation, and gluconeogenesis, respectively. Indeed, SREBP1c is pivotal in lipid synthesis and metabolism, and its dysregulation is often linked to fatty liver disease [38]. Similarly, CPT1A is essential for fatty acid oxidation [39], while PEPCK is a key enzyme in gluconeogenesis [40]. These genes are often dysregulated in liver diseases such as fatty liver and metabolic syndrome. FOH’s ability to modulate these pathways (as demonstrated in the current study) suggests its potential in reestablishing hepatic energy homeostasis, which is disrupted by TM-induced ER stress. This finding is consistent with previous research highlighting the importance of metabolic balance in liver function [41].

Furthermore, FOH alleviates oxidative stress induced by TM, as evidenced by reduced levels of MDA (a marker of lipid peroxidation) and restored antioxidant indices like SOD and GSH. These findings underscore FOH’s antioxidative properties and its role in maintaining redox homeostasis, which is disrupted by TM. These findings align with prior research that emphasizes FOH’s role in reducing oxidative stress [20,42]. Histological analysis further supports these observations, showing that FOH treatment mitigates lipid droplet accumulation and restores liver architecture, which is compromised by TM-induced injury. These results align with studies that highlight FOH’s hepatoprotective effects, particularly in the context of oxidative stress and inflammation [18,34]. For instance, Abukhalil, Hussein [18], who investigated the protective effects of farnesol (FOH) against liver injury in rats fed a high cholesterol diet (HCD), demonstrated that FOH significantly reduced serum lipids, liver cholesterol, and triglyceride levels, while also improving liver function markers.

In vivo, FOH treatment also restores lipid metabolism, further emphasizing its role in restoring metabolic balance during ER stress. The regulation of these metabolic processes is critical, as dysregulated lipid and glucose metabolism can lead to liver diseases such as steatosis and metabolic syndrome. Our findings also show that FOH stabilizes serum lipid profiles, including triglycerides and cholesterol levels, even though changes in ALT and AST levels were not statistically significant. TM treatment led to the upregulation of genes linked to lipid accumulation and gluconeogenesis, indicative of metabolic dysfunction. FOH treatment effectively modulated these gene expressions, suggesting its role in restoring metabolic balance and preventing lipid dysregulation, which can lead to hepatic steatosis and liver disease [18]. The current results suggest that FOH may offer therapeutic potential in normalizing serum lipid profiles under stress, providing a non-invasive approach to managing liver dysfunction. These results are aligned with the findings of Pant and Rondini [43], who demonstrated that FOH promotes fatty acid oxidation and reduces triglyceride accumulation. Additionally, some studies [44,45] corroborate our findings, showing that FOH improves glycogen storage and reduces triglyceride levels both in liver tissues and serum. Previous literature further supports the potential of FOH in ameliorating dyslipidemia and improving liver function by reducing serum inflammatory markers and preventing hepatic lipid infiltration [18,46,47]. Together, these studies strengthen the therapeutic promise of FOH in addressing metabolic disturbances and liver dysfunction associated with ER stress.

Lastly, our animal model showed no significant effects of FOH on body weight or fat tissue, suggesting that FOH protects against TM-induced liver dysfunction without altering overall body mass. This is consistent with previous studies [48,49] demonstrating FOH’s ability to protect against lipid accumulation in the liver, without affecting body weight or fat distribution.

While this study provides key insights into the therapeutic potential of farnesol (FOH) in mitigating oxidative stress and restoring metabolic homeostasis during ER stress, a more comprehensive analysis of the unfolded protein response (UPR) branches (such as PERK, eIF2α, ATF6, and IRE1α) could enhance understanding of FOH’s mechanism. Although additional UPR markers could offer deeper mechanistic insights, the present study successfully demonstrates FOH’s protective effects against TM-induced oxidative damage, metabolic disruptions, and liver dysfunction, thus establishing its potential as a non-invasive therapeutic agent for acute liver stress. This work paves the way for future research on the molecular mechanisms of FOH, particularly in treating metabolic diseases linked to ER stress.

## 5. Conclusions

This study demonstrates the therapeutic potential of farnesol (FOH) in alleviating oxidative stress and restoring metabolic homeostasis in liver cells under tunicamycin (TM)-induced ER stress. FOH effectively mitigates oxidative damage, reduces key ER stress markers such as ATF4, and restores metabolic pathways critical for lipid and glucose metabolism, thereby protecting liver function and stabilizing serum lipid profiles. These findings suggest FOH as a promising non-invasive treatment for liver dysfunction and metabolic diseases linked to ER stress. However, while our study highlights significant gene expression changes and metabolic improvements following FOH treatment, further validation through Western blot analysis is recommended to confirm the modulation of key proteins involved in ER stress and metabolic pathways. Additionally, a more comprehensive exploration of other unfolded protein response (UPR) branches, including PERK, eIF2α, ATF6, and IRE1α, would enhance the understanding of FOH’s broader molecular mechanisms, providing stronger evidence for its therapeutic potential.

## Data Availability

The data presented in this study are available on request from the corresponding author.

## Supplementary Materials

The following supporting information can be downloaded at https://www.mdpi.com/article/10.3390/biology14020213/s1. Table S1 Main reagents used in this study. Table S2 Main instruments used in this study. Table S3: qRT-PCR preparation. Table S4: qRT-PCR program. Table S5: Sequences of primers in this study. Supplementary Figure S1. Effect of farnesol on body weight & epididymal fat weight among all groups of mice. (A) Body weight. (B) Epididymal fat weight. Values represent the mean ± SEM from three independent biological replicates.
