# Supplementary material for: Farnesol Improves Endoplasmic Reticulum Stress and Hepatic Metabolic Dysfunction Induced by Tunicamycin in Mice
Source: Biology (Basel). 2025 Feb 18;14(2):213. doi: 10.3390/biology14020213 (PMC11851907; doi:10.3390/biology14020213)
Supplement: Supplementary file 1 [file biology-14-00213-s001.zip › biology-3333291-supplementary.pdf]

Farnesol Improves Endoplasmic Reticulum Stress and Hepatic Metabolic Dysfunction Induced by Tunicamycin in Mice

Supplementary Materials

Table S1 Main reagents used in this study

Reagents	Company	Supplier
**Tunicamycin**	Sigma-Aldrich	Germany
**Farnesol**	Sigma –Aldrich	Germany
**PBS**	Sigma –Aldrich	Germany
**DMSO**	Sigma –Aldrich	Germany
**DMEM**	Sigma –Aldrich	Germany
**Trizol**	Invitrogen	Shanghai China
**Chloroform**	Sigma -Aldrich	Germany
**Isopropanol**	Sigma -Aldrich	Germany
**RNase free water (DEPEC)**	Sango biotech	Shanghai China
**Takara RNase mini kit**	Takara	Dalian china
**PrimeScrip^{TM} RT reagent kit with gDNA Eraser 2xTaq Master Mix**	Takara	Dalian china
**Absolute ethyl alcohol**	Sigma -Aldrich	Germany
**Formalin**	Big sharp. China	Shanghai China

Table S2 Main instruments used in this study

Instrument	Company	Country origin
**Electronic balance**	Sartorius	Germany
**Constant temperature water bath**	HH-2	China
**Optical microscope**	Olympus,	Japan
**Digital imaging microscope**	TS100, Nikon,	Tokyo japan
**Refrigerator**	Haier	Shandong province, China
**Centrifuge**	TDZ4-ws	China
**Ultra-low temperature freezer**	ThermoFisher	USA
**Pcr cycler master**	BioSystems	USA
**Electrophoresis chamber**	Bio-rad power	USA
**Nanodrop one**	ThermoFisher scientific	USA
**Quantitative PCR amplification**	7900HT, ABI, Carlsbad, CA,	USA

Table S3: qRT-PCR preparation

qRT-PCR mix for one reaction Component	Volume
**SYBR Green Master Mix (2 ×)**	5 μl
**PCR Primer forward**	1μl
**PCR Primer reverse**	1μl
**cDNA sample**	3.0μl
**Final volume**	10 μl

Table S4: qRT-PCR program

Quantitative real-time PCR Step	Cycles	Temperature (°C)	Time
**Dissociation**	1	95	10 second
**Amplification**			
**Dissociation**		95	10
**Annealing**		60	10
**Elongation**	40	72	20
**Cooling**	1	12	30

Table S5: Sequences of primers in this study

**β-actin**	**F5'-GGCTGTATTCCCCTCCATCG-3'**<br>**R 5'-CCAGTTGGTAACAATGCCATGT-3'**	**NM_007393.5**
**Chop**	F5’-CACGCACATCCCAAAGCC-3’<br>R5’-GGGCACTGACCACTCTGTT-3’	NM_007837
**Grp78**	F5’-ATCAGGGCAACCGCATCAC-3<br>R5’-TGATGTCCTGCTGCACCGAA-3	NM_001163434
**Atf4**	F5’-TGACTTCGATGCTCTGTTTCGA-3’<br>R5’-CCAACGTGGTCAAGAGCTCAT-3’	NM_009716
**Pepck**	F5'-CGCTGGATGTCGGAAGAGG-3'<br>R 5'-GGCGAGTCTGTCAGTTCAATAC-3'	NM_011044.2
**Scd1**	F5'-CCTACGACAAGAACATTCAATCCC-3'<br>R 5'-CAGGAACTCAGAAGCCCAAAGC-3'	NM_009127.4
**Fas**	F5'-GGCTCTATGGATTACCCAAGC-3'<br>R 5'-CCAGTGTTCGTTCCTCGGA-3'	NM_007988.3
**G6pase**	F5'-CGACTCGCTATCTCCAAGTGA-3'<br>R 5'-GTTGAACCAGTCTCCGACCA-3'	NM_008061.4
**Srebp1c**	F’5-AACTGCCCATCCACCGACTC-3’<br>R5’-ATTGATAGAAGACCGGTAGCGC-3’	NM_001313979

**Cpt1a**	F5'-CTCCGCCTGAGCCATGAAG-3<br>R 5'-CACCAGTGATGATGCCATTCT-3'	NM_013495.2

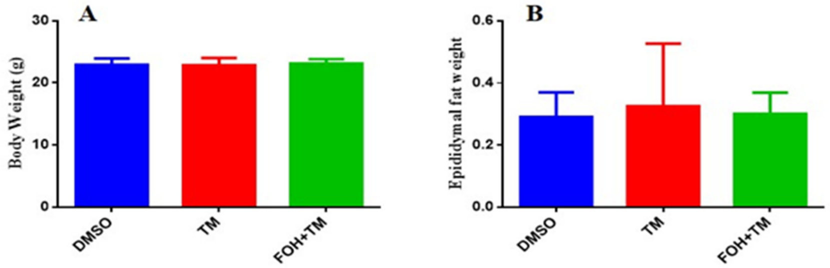

Supplementary Figure S1. Effect of farnesol on body weight & epididymal fat weight among all groups of mice. (A) Body weight. (B) Epididymal fat weight. Values represent the mean ± SEM from three independent biological replicates.
